# Social Perspective Taking Is Associated With Self-Reported Prosocial Behavior and Regional Cortical Thickness Across Adolescence

**DOI:** 10.1037/dev0000541

**Published:** 2018-07-30

**Authors:** Christian K. Tamnes, Knut Overbye, Lia Ferschmann, Anders M. Fjell, Kristine B. Walhovd, Sarah-Jayne Blakemore, Iroise Dumontheil

**Affiliations:** 1 Department of Psychology, University of Oslo; 2 Center for Lifespan Changes in Brain and Cognition, Department of Psychology, University of Oslo; 3 Institute of Cognitive Neuroscience, University College London; 4 Department of Psychological Sciences, Birkbeck, University of London

**Keywords:** brain structure, development, MRI, social cognition, theory of mind

## Abstract

Basic perspective taking and mentalizing abilities develop in childhood, but recent studies indicate that the use of social perspective taking to guide decisions and actions has a prolonged development that continues throughout adolescence. Here, we aimed to replicate this research and investigate the hypotheses that individual differences in social perspective taking in adolescence are associated with real-life prosocial and antisocial behavior and differences in brain structure. We used an experimental approach and a large cross-sectional sample (*n* = 293) of participants aged 7–26 years old to assess age-related improvement in social perspective taking usage during performance of a version of the director task. In subsamples, we then tested how individual differences in social perspective taking were related to self-reported prosocial behavior and peer relationship problems on the Strengths and Difficulties Questionnaire (*n* = 184) and to MRI measures of regional cortical thickness and surface area (*n* = 226). The pattern of results in the director task replicated previous findings by demonstrating continued improvement in use of social perspective taking across adolescence. The study also showed that better social perspective taking usage is associated with more self-reported prosocial behavior, as well as to thinner cerebral cortex in regions in the left hemisphere encompassing parts of the caudal middle frontal and precentral gyri and lateral parietal regions. These associations were observed independently of age and might partly reflect individual developmental variability. The relevance of cortical development was additionally supported by indirect effects of age on social perspective taking usage via cortical thickness.

Fundamental aspects of perspective taking emerge in the second year of life (Moll & Tomasello, 2006; Sodian, Thoermer, & Metz, 2007), whereas theory of mind or *mentalizing*, the ability to attribute mental states such as beliefs, desires and intentions, is thought to develop in early childhood. Classic theory of mind tasks that examine the explicit understanding of other people’s false beliefs or second-order beliefs are typically passed around age 4 and 7 years, respectively (Perner & Wimmer, 1985; Wellman, Cross, & Watson, 2001). More recent studies however, indicate that the use of *social perspective taking* (i.e., the ability to take the perspective of another individual into account in a communicative context and use this information to guide decisions and actions) continues to develop across childhood and adolescence (Begeer et al., 2016; Dumontheil, Apperly, & Blakemore, 2010; Mills, Dumontheil, Speekenbrink, & Blakemore, 2015; Symeonidou, Dumontheil, Chow, & Breheny, 2016). In the present study, we aimed to replicate this finding and to extend our understanding of social perspective taking across multiple levels of individual differences data by relating it to real-life social behavior, on the one hand, and cerebral cortex structure, on the other.

Prolonged development of social perspective taking has been found in studies using variants of the director task (Apperly et al., 2010; Keysar, Barr, Balin, & Brauner, 2000; Keysar, Lin, & Barr, 2003). This is an experimental paradigm in which participants view sets of shelves containing objects, which they are instructed to move by an avatar (the “director”) who can see some but not all of the objects. Correct interpretation of critical instructions in the experimental condition requires participants to use the director’s perspective and to move only objects that the director can see. In a control condition, participants are asked to ignore certain objects according to a simple visual rule, specifically to move objects only in clear shelf slots and ignore objects in slots with a gray background. Dumontheil et al. (2010) tested a large sample of female participants in the age range 7–27 years using this task and found that accuracy in the perspective-taking condition continued to improve between adolescence and adulthood. As successful perspective taking in this task also involves inhibiting one’s own perspective and integrating one’s goals with the context, this prolonged development might reflect interactions between perspective taking and developing executive functions (Dumontheil, Apperly, et al., 2010). Two smaller studies using versions of the director task have replicated the finding of continued development of social perspective taking usage across adolescence (Humphrey & Dumontheil, 2016; Symeonidou et al., 2016).

In summary, several studies have demonstrated that, contrary to earlier assumptions that mentalizing stops developing in early childhood, the ability to use someone else’s perspective to guide ongoing behavior is still developing throughout adolescence. A next step in this theoretical framework is to ask how perspective taking, as measured in a lab-based experimental task, relates to real world social behavior in adolescence. As social perspective taking is necessary to understand that someone else might think and feel differently than you do, it is thought to be a key building block of both sympathy and empathy, and to in turn foster prosocial behavior (Decety, Bartal, Uzefovsky, & Knafo-Noam, 2016). Conversely, poor social perspective taking ability might lead to social maladjustment and peer problems. In a recent study, Sierksma, Thijs, Verkuyten, and Komter (2014) interviewed children about helping situations in vignettes that varied in the recipient’s need for help and in the costs to the helper. The results showed that, when both need and costs were high, social perspective taking ability, measured via a separate task requiring understanding of a false evaluation of another character, was positively associated with stronger moral indignation against a character refusing to help another. There is a large body of evidence for a small positive association between children’s theory of mind scores and concurrent measures of prosocial behavior, and this association appears to hold for both cognitive and affective theory of mind and for different subtypes of prosocial behavior (Imuta, Henry, Slaughter, Selcuk, & Ruffman, 2016). Longitudinal studies with children additionally support a mediational hypothesis of an indirect path from theory of mind to subsequent lower peer rejection and higher peer acceptance, via improvements in prosocial behavior (Caputi, Lecce, Pagnin, & Banerjee, 2012), and suggest that aspects of theory of mind performance inversely predict later reactive and proactive aggression (Austin, Bondü, & Elsner, 2017). A hypothesis tested in the current study is that individual differences in social perspective taking usage will be associated with individual differences in real-life pro- and antisocial behavior in adolescence, a period of life when our social world becomes more complex and we hone our skills at navigating increasingly manifold and intimate relationships (Blakemore & Mills, 2014). Two previous experimental studies have shown links between social perspective taking and behavioral measures of social behavior. Specifically, these studies found associations between performance on the director task and trust and reciprocity toward others in the trust game (Fett et al., 2014), and between self-reported perspective-taking skills and age-related increases in noncostly prosocial behavior toward friends (Güroğlu, van den Bos, & Crone, 2014), respectively. However, less is known about how adolescents’ social perspective taking, as measured experimentally by the director task, relates to naturally occurring social behavior, which we aimed to investigate in the current study.

The next aim of the current study was to investigate how individual differences in social perspective taking usage relate to individual difference in brain structure. Prolonged development of use of social perspective taking is consistent with neuroimaging studies of brain development. Both structural (Giedd et al., 2015; Mills, Lalonde, Clasen, Giedd, & Blakemore, 2014; Tamnes et al., 2013) and functional (Blakemore & Robbins, 2012; Braams & Crone, 2017; Flannery, Giuliani, Flournoy, & Pfeifer, 2017; Flournoy et al., 2016; Luna, Padmanabhan, & O’Hearn, 2010; Overgaauw, van Duijvenvoorde, Gunther Moor, & Crone, 2015; Sebastian et al., 2012) MRI studies indicate that brain regions critically involved in social cognition, including dorso-medial prefrontal and lateral temporo-parietal cortices, and/or executive functions, including lateral prefrontal and anterior cingulate cortices, show protracted developmental changes. For example, toward the end of the teenage years, cortical gray matter volume reductions exceeding the average rate are seen primarily in medial prefrontal, lateral prefrontal and lateral temporo-parietal regions (Tamnes et al., 2013). Surface-based cortical reconstruction software also allows for the ability to measure not only cortical volume, but also its separate components thickness and surface area (Dale, Fischl, & Sereno, 1999; Fischl, Sereno, & Dale, 1999). *Thickness* is defined as the estimated distance between the outer and inner boundary of the cortical sheet and area is defined as the estimated expansion or contraction of points on the surface (Mills & Tamnes, 2014). Although it is believed that, at birth, cortical surface area is largely determined by the number of cortical columns and cortical thickness by the number of cells within a column (Geschwind & Rakic, 2013), the biological processes that drive later individual and developmental differences are not known. However, longitudinal studies document that these distinct structural properties show unique developmental trajectories across different stages of life (Lyall et al., 2015; Storsve et al., 2014), including across adolescence (Raznahan et al., 2011; Tamnes et al., 2017; Vijayakumar et al., 2016; Wierenga, Langen, Oranje, & Durston, 2014). Although some disagreements exist between available studies regarding the precise development across adolescence, a recent multisite study, which included four independent longitudinal data sets, showed consistent, widespread, and regionally variable nonlinear decreases in cortical thickness and comparably smaller steady decreases in surface area (Tamnes et al., 2017).

An increasing number of studies address the cortical foundations of cognitive development (Jernigan, Brown, Bartsch, & Dale, 2016; Walhovd, Tamnes, & Fjell, 2014). However, studies investigating associations between brain structure and social cognition during childhood and adolescence are scarce, and only a limited number of studies have linked individual differences in brain structure to individual differences in social cognition in adults (Kanai & Rees, 2011). A small number of studies have used social network size (e.g., on Facebook) as a proxy for assessing social–cognitive functioning and have found associations with the size of the amygdala (Von Der Heide, Vyas, & Olson, 2014) and temporal cortex in adults (Kanai, Bahrami, Roylance, & Rees, 2012). Another study found that anthropomorphic attribution was associated with gray matter volume in the left temporo-parietal junction in adults (Cullen, Kanai, Bahrami, & Rees, 2014). Here, we investigate the relations between individual differences in social perspective taking usage and brain structure in adolescence, to improve our understanding of the sources of variation in social cognition during this period of development.

In the present study, we aimed to (a) test the reproducibility of the previously reported pattern of age-related improvements in use of social perspective taking across adolescence (Dumontheil, Apperly, et al., 2010; Humphrey & Dumontheil, 2016; Symeonidou et al., 2016); (b) investigate the relationship between individual differences in social perspective taking usage and self-reported real-life social behavior; (c) investigate the relationship between individual differences in social perspective taking usage and structure of the cerebral cortex. We hypothesized that social perspective taking usage would show continued age-related improvement across adolescence. We also predicted that better social perspective taking, independent of age, would be associated with more reported prosocial behavior and fewer reported peer relationship problems, as well as with relatively more mature cortical structure, that is, lower thickness and possibly lower surface area, in regions involved in mental state attribution and/or executive functions. These predictions were based on the idea that age-independent associations reflect, at least to some extent, individual developmental variability (Jernigan, Baaré, Stiles, & Madsen, 2011).

## Materials and Methods

### Participants

Participants aged 7–26 years were recruited through advertisements and local schools in Oslo, Norway, and originally participated in in one of two longitudinal projects—Neurocognitive Development (Tamnes et al., 2010) or the Norwegian Mother and Child Cohort Neurocognitive Study (Krogsrud et al., 2014)—or a student research project. Written informed consent was obtained from a parent of all participants under 16 years of age and from participants 12 years of age and older, whereas participants under 12 years of age gave oral assent. The Regional Committee for Medical and Health Research Ethics Norway approved the study (2009/200: Nevrokognitiv utvikling i skolealder—oppfølgingsstudie).

Participants aged 16 years or older, and parents of participants under 16 years, completed standardized health interviews regarding each participant to ascertain eligibility. All participants were required to be fluent Norwegian speakers, have normal or corrected-to normal vision and hearing, not have any injury or disease known to affect central nervous system function, including diagnosed neurological or psychiatric illness or serious head trauma, and not use psychoactive drugs known to affect central nervous system functioning.

Three hundred and two participants satisfied these criteria. Nine participants were excluded based on behavioral criteria defined in the director task, as described below. This yielded a sample of 293 participants (164 females) aged 7.1–26.7 years (*M* = 16.9, *SD* = 5.1). The age for females (*M* = 16.9 years, *SD* = 5.3) and males (*M* = 16.9 years, *SD* = 4.8) was not significantly different (*t* = 0.03, *p* = .980). The sample had a mean IQ of 110.1 (*SD* = 11.2, range = 79–141, missing data for 23 participants) as estimated by a Norwegian version of the Wechsler Abbreviated Scale of Intelligence two-subtest form (Wechsler, 1999), including the Vocabulary and Matrix Reasoning subtests. Age groups were created to allow for direct comparison with previous studies reporting on age-related differences in performance on versions of the director task (Dumontheil, Apperly, et al., 2010; Symeonidou et al., 2016): children (*n* = 60, 7.1–11.3 years, 38 females), adolescents (*n* = 108, 11.7–17.9 years, 53 females), and adults (*n* = 125, 18.0–26.7 years, 73 females).

Of the 293 participants in the full sample, 184 (63%, 101 females) were included in the analyses testing for associations between director task performance and self-reported behavior, as measured by the Strengths and Difficulties Questionnaire (SDQ; 63 were below 12 years old and were not asked to complete the self-report SDQ, 21 older adolescents participated in a student research project where SDQ was not include in the protocol, and 25 had missing data). The participants in the SDQ sample were 12.2–26.1 years (*M* = 18.9, *SD* = 3.4).

Finally, of the 293 participants in the full sample, 226 (77%, 122 females) were included in the analyses testing for associations with structural properties of the cerebral cortex (40 children were part of a research project that used a different MRI protocol [Krogsrud et al., 2014] and were thus not included, 21 older adolescents participated in a student research project that did not include scanning, and six were excluded during post-processing quality control as described below). The resulting MRI sample was aged 8.5–26.7 years (*M* = 18.3, *SD* = 4.2).

### Experimental Task

Social perspective taking usage was assessed by a version of the director task, originally adapted from Keysar et al. (Keysar et al., 2000, 2003) by Apperly et al. (2010), and modified for the present study. E-Prime 2.0 (Psychology Software Tools, Pittsburgh, PA) was used for stimulus presentation and response logging. All participants first carried out the experimental director condition before the control no-director condition of the task.

**Figure 1 fig1:**
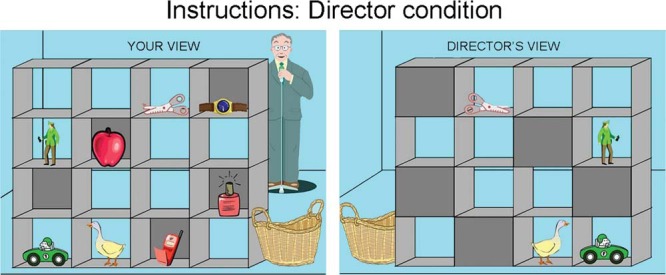
Director task instructions. Before the experimental director condition of the task, participants were shown and explained images of their view and the corresponding director’s view of a stimulus configuration with an example of an object that both the participant and the director can see (“car”), and an example of an object that the participant, but not the director, can see (“apple”).

Standardized instructions were read to the participants before each condition. For the director condition (Figure 1), participants were shown an example stimulus. It was explained that, on each trial, they will be shown a set of shelves containing various objects in different slots and that the man standing on the other side of the shelves (the “director”) will ask the participant to move specific objects to the basket. Emphasis was placed on the fact that the director had a different perspective to the participant, by explaining and showing that some of the slots are occluded and that the participant, but not the director, can see the objects in these slots. Participants were shown an illustration of the director’s view of the same stimulus and it was reiterated that the director cannot see the objects in the occluded slots and that the participant will have to think about this when performing the task. The task administrator then showed an example of an object that both the participant and the director could see (“car”), and an example of an object that the participant, but not the director, could see (“apple”). The participant was then asked to give a different example of an object that only she or he, and not the director, could see, and an object that both could see. Instructions were repeated if needed. Participants were asked to respond as accurately and quickly as possible by pointing and clicking with the computer mouse and were then given three practice trials.[Fig-anchor fig1]

**Figure 2 fig2:**
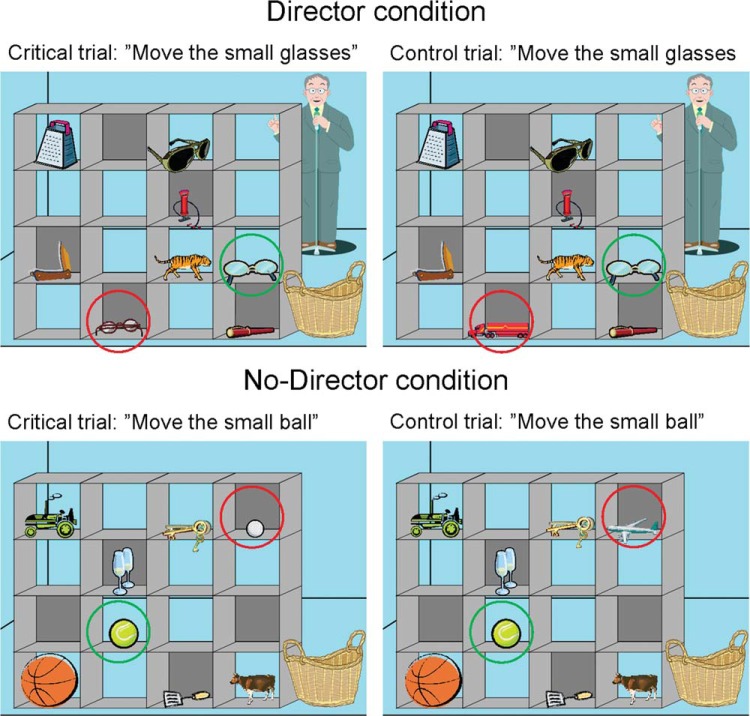
Director task. Top panel: Examples of a critical trial and a control trial in the experimental director condition. Bottom panel: Examples of a critical trial and a control trial in the control no-director condition. For illustration purposes, the target stimulus is highlighted with a green (light gray) circle, whereas the distractor/irrelevant object is highlighted with a red (dark gray) circle.

In critical trials during the experimental director condition, participants were required to take account of the director’s perspective and the correct response was to select the target object, which could be seen by the director, and was the best fit for his instruction if his visual perspective was taken into account. For example, in Figure 2’s top left panel when the director asks, “move the small glasses,” the correct response would be to move the glasses with the yellow frame, that is, the second smallest glasses. If participants ignored the director’s perspective they would select the distractor object, the glasses with the red frame, which were the smallest in the shelves but not visible to the director. In control trials, the arrangement of the objects in the shelves was identical to that in the critical trials, except that an irrelevant object replaced the distractor object (e.g., the truck in Figure 2’s top right panel). In filler trials, instructions referred only to objects in clear slots, that is, visible to both the participant and the director (e.g., “move the tiger”).[Fig-anchor fig2]

Before the start of the control no-director condition, new instructions were read while participants were shown two examples of stimuli without the director on the other side of the shelves present. It was explained that some slots in the shelves have gray back panels, whereas others are clear, and that the participant in each trial will be asked to move specific object to the basket, but that they should only move objects in clear slots. It was stressed that the participant should ignore objects in the slots with a gray background. Each participant was asked to give examples of objects in both types of slots and was then asked to select an object as they would in a critical trial to demonstrate that they understood what was required of them. The no-director trials were identical in every way to the director trials except that the director on the other side of the shelves was not present, and instead of having to take into account the director’s perspective, participants were instructed to follow the rule of ignoring all objects in slots with a gray background. Critical, control and filler trials were included in the no-director condition. For example, in Figure 2’s lower left panel when instructed to “move the small ball,” the correct response would be to select the second smallest ball, the yellow ball, and ignore the distractor object, the white ball, which was the smallest ball in the shelves, but was in a slot with a gray background. In control trials, an irrelevant object replaced the distractor object (e.g., the airplane in Figure 2’s lower right panel). Thus, critical trials of both the director and the no-director condition involved inhibition of a prepotent response of moving the object that best fit the instruction from the participant’s perspective, as well as general task demands. The conditions critically differed in whether the participants were instructed to consider another’s perspective or to follow a simple visual rule. Control trials included the requirements of critical trials to process relative size or position information from an auditory instruction but did not require participants to take into account the perspective of the director or inhibit a dominant response. Filler trials served to reduce the saliency of the key trials of interest.

For each participant, the version of the director and no-director conditions administered were randomly selected from six alternative versions to counterbalance the order of different trial types and stimuli configurations across participants. In both conditions, participants were shown on the computer screen cartoon pictures of a 4 × 4 set of shelves containing eight different objects and five slots with gray backgrounds (occluded slots). Each shelf-object configuration was first presented for 2 s, and then three successive auditory instructions were presented, corresponding to two filler trials and one control trial or two filler trials and one critical trial. Each of these trials lasted for 6 s. The instructions were played through the computer speakers and asked the participant to move one of the eight objects, either by only referring to the object name (Filler trials) or by the object name in combination with size (small/large) or relative horizontal position (top/bottom) information (control and critical trials). Compared to the developmental study by Dumontheil et al. (2010), where the instructions in the task asked the participants to move specific objects left, right, up or down, our modified version of the task only asked participants to move the object into a basket by clicking it. Thus, the aspect of the task requiring directional decisions, a potential confound, was eliminated. In total, there were eight critical trials, eight control trials, and 32 filler trials in each condition (director and no-director). Each condition lasted approximately 5.5 min.

Behavioral criteria were used to exclude participants with performance indicating that they had not understood the instructions of the task or had suboptimal motivation or task focus. Specifically, participants with 0% accuracy for any trial type in either of the two conditions were excluded. This led to the exclusion of nine participants, seven of whom were excluded based on performance on critical trials in the experimental director conditions, and two of which were excluded based on performance on critical trials in the control no-director condition. All behavioral data reported are based on the remaining 293 participants. Accuracy (percentage errors) and intraindividual median response times (RTs) in correct trials were calculated for each participant in each condition (director/no-director) and trial type (critical/control/filler). In addition, we computed the difference in percentage errors on director critical trials and on no-director critical trials, to be used in the analyses testing for associations with self-reported behavior and cortical structure, as described below.

### Behavioral Questionnaire

The SDQ self-report version was used to assess participants’ behavior (R. Goodman, Meltzer, & Bailey, 1998). The SDQ is a well-validated and clinically broadly used questionnaire which asks about 25 attributes, rated on a 3-point Likert scale, equally divided between five scales: emotional symptoms, conduct problems, hyperactivity/inattention, peer relationship problems, and prosocial behavior. Recent studies indicate that SDQ is not only suitable for distinguishing clinical and healthy groups of children but is also a valid continuous measure of child and adolescent mental health across the full range of variation (A. Goodman & Goodman, 2009). For the current study, we used only the prosocial behavior and peer relationship problems scales.

### Image Acquisition

MRI acquisition was done with a 3.0T Siemens Skyra (Erlangen, Germany) with a 24-channel coil. Three-dimensional T1-weighted MP-RAGE sequences with the following parameters were used for volumetric and cortical surface analyses: repetition time = 2,300 ms; echo time = 2.98 ms; inversion time = 850 ms; flip angle = 8°; bandwith = 240 Hz/pixel; field of view = 256 mm; and scan time = 9:50 min. The sequence consisted of 176 sagittal slices with a voxel size of 1.0 × 1.0 × 1.0 mm.

### Image Processing

Volumetric segmentation and cortical reconstruction and was performed with the FreeSurfer image analysis suite Version 5.3, which is documented and freely available for download online (http://surfer.nmr.mgh.harvard.edu/). The technical details of these procedures are described in prior publications (Dale et al., 1999; Fischl et al., 1999, 2002, 2012). Briefly, the processing includes motion correction, removal of nonbrain tissue, automated Talairach transformation, segmentation of subcortical volumetric structures, intensity normalization, tessellation of surfaces, automated topology correction, and surface deformation to optimally place tissue borders. Cortical thickness maps for each subject were obtained by calculating the distance between the cortical gray matter and white matter surface at each vertex (surface point; Fischl & Dale, 2000). Cortical surface area (white matter surface) maps were computed for each subject by calculating the area of every triangle in the tessellation. The triangular area at each location in native space was compared with the area of the analogous location in registered space to give an estimate of expansion or contraction continuously along the surface (“local arealization;” Fischl et al., 1999). The maps produced are not restricted to the voxel resolution of the original data and are thus capable of detecting submillimeter differences. In addition to screening of all images immediately after data acquisition and rescanning if needed and possible, all processed scans were visually inspected in detail as part of the quality control procedure. Before statistical analyses, surface maps for cortical thickness and area were smoothed with a Gaussian kernel of full-width at half maximum of 15 mm.

### Statistical Analysis

For the full sample (*n* = 293, 7.1–26.7 years), participants on average made only 1.6% and 1.4% errors in filler trials in the director and the no-director condition, respectively, and the data from these trials were not included in further analyses. For both accuracy and RT, a 2 × 2 × 3 mixed analysis of variance (ANOVA) with condition (director, no-director) and trial type (critical, control) as within-subject factors and age group (children, adolescents, adults) as between-subjects factors was performed. ANOVAs on separate trial types, conditions or age groups and independent samples *t* tests between age groups were performed as follow-up analyses to further investigate significant interaction and main effects.

We then tested for associations between performance on the director task and both self-reported behavior and structure of the cerebral cortex. Our task performance measure of interest for these analyses was the difference in percentage errors on director critical trials and on no-director critical trials. This measure was chosen in order to identify individual differences in social perspective taking, while controlling for some general and executive function task demands. First, for a subsample of adolescents and young adults (*n* = 184, 12.2–26.1 years), we used general linear models (GLMs) in SPSS with each of the two SDQ scales of interest (prosocial behavior, peer relationship problems) as the dependent variable, sex as a fixed factor, and age and task performance as covariates.

Second, for the MRI sample (226 participants, 8.5–26.7 years old), we performed surface-based cortical analyses on a vertex-wise (point-by-point) level using GLMs as implemented in FreeSurfer. Effects of task performance on both cortical thickness and surface area were tested. Initially, this was done while controlling only for sex to test for temporal co-occurrence of overall developmental trends in behavior and cortical structure. Such associations do however not necessarily imply that the variables are directly interrelated (Salthouse, 2011). The analyses were therefore then repeated while additionally controlling for age, as it is reasonable to hypothesize that such age-independent associations are mediated, at least to some extent, by developmental variability, that is, variability among adolescents of similar age in the phase of brain maturation (Jernigan et al., 2011). The data were tested against an empirical null distribution of maximum cluster size across 10,000 iterations using Z Monte Carlo simulations as implemented in FreeSurfer (Hagler, Saygin, & Sereno, 2006; Hayasaka & Nichols, 2003) synthesized with a cluster-forming threshold of *p* < .05, yielding clusters fully corrected for multiple comparisons across the surfaces. Clusterwise corrected *p* < .05 was regarded as significant. Mean cortical thickness was then extracted from each significant cluster and we performed GLMs in SPSS with thickness as dependent variable, sex as fixed factor, and age and task performance as covariates to obtain effect size estimates. Note however that these are inflated because they are based on already-identified significant clusters. To test the indirect effect of age on director task performance through cortical thickness, Hayes’ PROCESS tool was used (v2.16.3; mediation model number 4; 10,000 bootstrap resamples; Hayes, 2013). An indirect path is considered statistically significant if the associated 95% confidence interval (CI) does not include zero.

## Results

### Director Task: Accuracy

**Figure 3 fig3:**
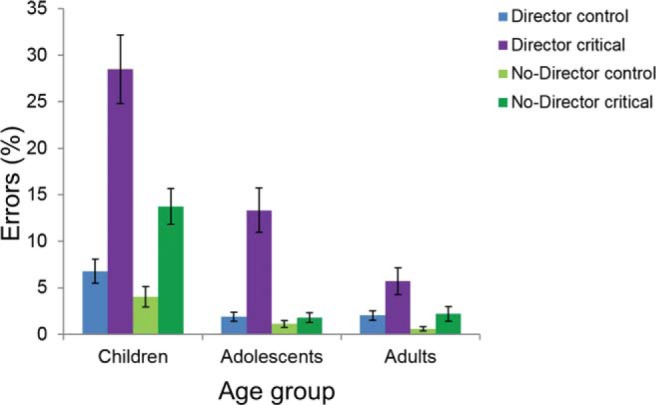
Director task performance: accuracy. Percentage errors (mean and standard errors) in control trials and critical trials in the director condition and the no-director condition for each age group. Children: 7.1–11.3 years (*n* = 60), adolescents: 11.7–17.9 years (*n* = 108), and adults: 18.0–26.7 years (*n* = 125).

A summary of task performance for the full sample is presented in Table 1. Average percentage errors for Critical trials and Control trials in the Director condition and the No-Director condition, respectively, for each of the three age groups are shown in Figure 3. All main effects were significant in a 2 (condition: director, no-director) × 2 (trial type: critical, control) × 3 (age group: children, adolescents, adults) mixed ANOVA on accuracy. Participants made more errors in the director condition than in the no-director condition, *F*(1, 290) = 69.63, *p* < .001, η_*p*_^2^ = .194, more errors on critical trials than on control trials, *F*(1, 290) = 95.76, *p* < .001, η_*p*_^2^ = .248, and accuracy differed with age group, *F*(2, 290) = 42.90, *p* < .001, η_*p*_^2^ = .228. There was a significant interaction between condition and trial type, *F*(1, 290) = 35.46, *p* < .001, η_*p*_^2^ = .109, between condition and age group, *F*(2, 290) = 6.88, *p* = .001, η_*p*_^2^ = .045, and between trial type and age group, *F*(2, 290) = 18.95, *p* < .001, η_*p*_^2^ = .116. The three-way interaction was also significant, *F*(2, 290) = 5.91, *p* = .003, η_*p*_^2^ = .039, and was explored further by looking at critical and control trials separately. [Table-anchor tbl1][Fig-anchor fig3]

A 2 × 3 mixed ANOVA performed on the critical trials showed main effects of condition, *F*(1, 290) = 58.34, *p* < .001, η_*p*_^2^ = .167, with more errors in the director condition, and age group, *F*(2, 290) = 33.03, *p* < .001, η_*p*_^2^ = .186, as well as a significant interaction between condition and age group, *F*(2, 290) = 7.14, *p* < .001, η_*p*_^2^ = .047. The same analysis on the control trials only showed significant main effects of condition, *F*(1, 290) = 11.48, *p* < .001, η_*p*_^2^ = .038, again with more errors in the director condition, and age group, *F*(2, 290) = 21.71, *p* < .001, η_*p*_^2^ = .130, but no significant interaction effect, *F*(2, 290) = 1.13, *p* = .323, η_*p*_^2^ = .008.

Follow-up analyses on the critical trials in the two conditions separately showed a significant effect of age group on accuracy in both the director condition, *F*(2, 290) = 20.88, *p* < .001, η_*p*_^2^ = .126, and the no-director condition, *F*(2, 290) = 36.42, *p* < .001, η_*p*_^2^ = .201. Independent samples *t* tests for the critical trials in the director condition revealed that the child group made significantly more errors than both the adolescent group (*t* = 3.45, *p* < .001, *d* = 0.66) and the adult group (*t* = 5.75, *p* < .001, *d* = 1.31), and also that the adolescent group made significantly more errors than the adult group (*t* = 2.73, *p* = .007, *d* = 0.41). Although for the critical trials in the no-director condition, the child group made significantly more errors than both the adolescent group (*t* = 5.99, *p* < .001, *d* = 1.45) and the adult group (*t* = 5.54, *p* < .001, *d* = 1.24), but there was no difference between the adolescent and the adult group (*t* = −0.41, *p* = .683). Additional analyses with age as a continuous variable showed very similar results (see the online supplementary material).

### Director Task: Response Times

**Figure 4 fig4:**
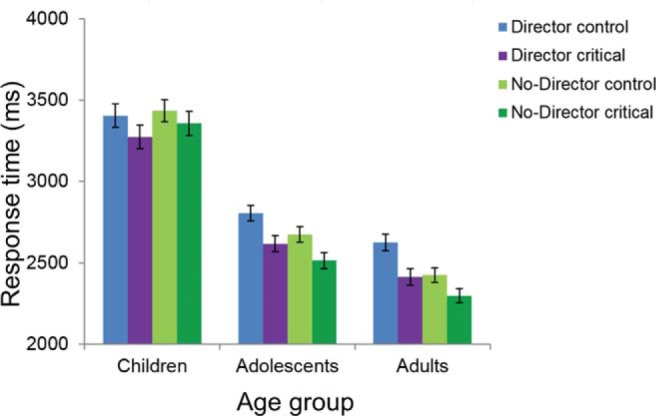
Director task performance: response time. Median response times (mean and standard errors) from correct trials only in control trials and critical trials in the director condition and the no-director condition for each age group. Children: 7.1–11.3 years (*n* = 60), adolescents: 11.7–17.9 years (*n* = 108), and adults: 18.0–26.7 years (*n* = 125).

Average median RTs for correct critical trials and control trials in the director condition and the no-director condition, respectively, for each of the three age groups are shown in Figure 4. All main effects were significant in a 2 (condition: director, no-director) × 2 (trial type: critical, control) × 3 (age group: child, adolescent, adult) mixed ANOVA on RT. Participants were slower overall in the director condition than in the no-director condition, *F*(1, 290) = 9.15, *p* = .003, η_*p*_^2^ = .031, and on control trials than on critical trials, *F*(1, 290) = 64.42, *p* < .001, η_*p*_^2^ = .182, and RTs differed with age group, *F*(2, 290) = 88.41, *p* < .001, η_*p*_^2^ = .379. There was a significant interaction between condition and age group, *F*(2, 290) = 6.27, *p* = .002, η_*p*_^2^ = .041, but no significant interactions between trial type and age group, *F*(2, 290) = 1.17, *p* = .312, η_*p*_^2^ = .008, or condition and trial type, *F*(1, 290) = 2.82, *p* = .094, η_*p*_^2^ = .010. The three-way interaction was not significant, *F*(2, 290) = 0.35, *p* = .708, η_*p*_^2^ = .002.[Fig-anchor fig4]

Because of the interaction between condition and age group, the main effect of condition on RTs was explored further in each age group separately. In the follow-up 2 × 2 mixed ANOVAs, the main effect of condition was not significant in the child group, *F*(1, 70) = 0.82, *p* = .368, η_*p*_^2^ = .014, but was significant in both the adolescent group, *F*(1, 96) = 10.23, *p* = .002, η_*p*_^2^ = .087, and the adult group, *F*(1, 124) = 25.35, *p* < .001, η_*p*_^2^ = .170. In both cases, participants were slower in the director condition than in the no-director condition (see the online supplementary material for analyses with age as a continuous variable).

### Associations Between Task Performance and Self-Reported Behavior

Relationships between director task performance and self-reported behavior on the SDQ were investigated with GLMs, with each of the two SDQ scales of interest (prosocial behavior, peer relationship problems) as dependent variable, sex as fixed factor, and age and the difference in percentage errors on director critical trials and on no-director critical trials as covariates. There was a significant small negative association between errors and prosocial behavior (*F* = 4.42, *p* = .037, η_*p*_^2^ = .024), such that participants who performed better on the Director task reported to show more prosocial behavior. In contrast, although there was a positive trend, the association between task errors and reported peer relationship problems was not significant (*F* = 2.83, *p* = .094, η_*p*_^2^ = .015).

### Associations Between Task Performance and Structure of the Cerebral Cortex

**Figure 5 fig5:**
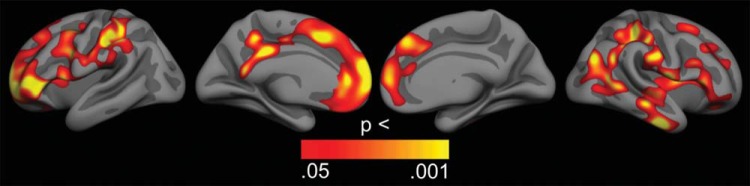
Associations between task performance and cortical thickness. general linear models (GLMs) were used to test the effects of the difference in percentage errors on director critical trials and on no-director critical trials on cortical thickness, while controlling for sex. The results were corrected for multiple comparisons using cluster size inference. Uncorrected *p* values within the corrected significant clusters are shown. All clusters showed positive effects, indicating that better performance was related to thinner cortices. No effects were seen in the opposite direction.

**Figure 6 fig6:**
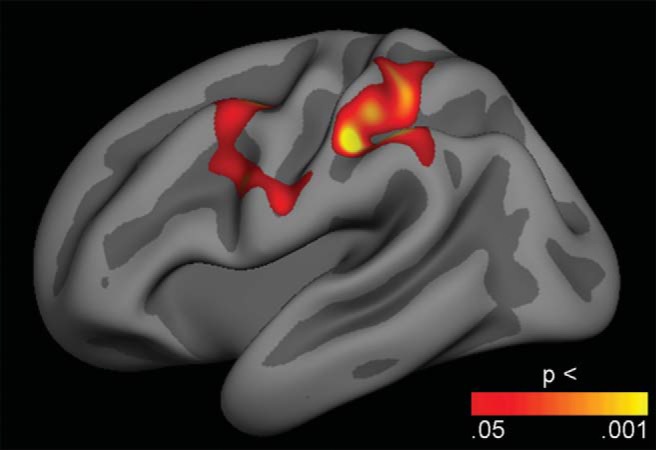
Age-independent associations between task performance and cortical thickness. General linear models were used to test the effects of the difference in percentage errors on director critical trials and on no-director critical trials on cortical thickness, while controlling for sex and age. The results were corrected for multiple comparisons using cluster size inference. Uncorrected *p* values within the corrected significant clusters are shown. Two clusters in the left hemisphere showed positive effects, indicating that better performance was related to thinner regional cortices. No effects were seen in the opposite direction.

Relationships between director task performance and cerebral cortex structure were initially explored across the cortical surface with GLMs testing the effects of the difference in percentage errors on director critical trials and on no-director critical trials on both cortical thickness and surface area, while controlling only for sex. After correction for multiple comparisons using cluster size inference, extensive bilateral fronto-parietal regions, including superior, lateral and medial prefrontal and lateral parietal cortices, as well as lateral temporal lobe regions in the left hemisphere showed positive associations between errors and cortical thickness (Figure 5). There were no significant effects in the other direction or on cortical surface area. We then repeated the analysis while additionally controlling for age, and these age-independent results showed two lateral regions in the left hemisphere with positive associations between errors and cortical thickness (Figure 6): A frontal cluster, which included parts of the caudal middle frontal and precental gyri (1,454 mm^2^, clusterwise *p* = .038, CI = .036 - .040), and a parietal cluster encompassing parts of the superior and inferior parietal lobules and the postcentral sulcus (1,997 mm^2^, clusterwise *p* = .006, CI = .005 - .007). These positive associations indicate that better performance was related to thinner cortices in these regions, independently of sex and age. Again, there were no significant effects in the other direction or on cortical surface area. To assess the size of the age-independent effects, we performed GLMs with mean cortical thickness in each of the two significant clusters as dependent variable, sex as fixed factor, and age and errors specifically on director critical trials as covariates. The results showed a small effect size for task performance in the frontal cluster (*F* = 4.21, *p* = .042, η_*p*_^2^ = .023) and a somewhat larger effect in the parietal cluster (*F* = 8.60, *p* = .004, η_*p*_^2^ = .046). [Fig-anchor fig5][Fig-anchor fig6]

Finally, the indirect effect of age on the difference in percentage errors between director critical trials and no-director critical trials via cortical thickness in the two identified clusters was tested in two mediation analyses using Hayes’ bootstrapping method. The analyses revealed significant indirect effects of age on director task performance via cortical thickness in the frontal cluster (indirect effect = −0.26, *SE* = 0.12, CIs = −0.552 to −0.089), and via cortical thickness in the parietal cluster (indirect effect = −0.35, *SE* = 0.13, CIs = −0.665 to −0.154), whereby older age was associated with lower cortical thickness in the two clusters, which was in turn associated with better director task perspective taking performance.

## Discussion

In this study, we tested for age-related differences in the ability to use information about another person’s perspective when following instructions and investigated whether this experimental measure of social perspective taking was related with self-reported real-life social behavior and with MRI-derived measures of the structure of the cerebral cortex. The behavioral results support previous findings of continued development of the use of social perspective taking across adolescence (Dumontheil, Apperly, et al., 2010). Further, independent of age, participants who performed better specifically on trials requiring social perspective taking reported more prosocial behavior and had thinner cerebral cortex in regions in the left hemisphere encompassing parts of the middle frontal gyrus and the lateral parietal lobe. There were also indirect effects of age on social perspective taking usage through cortical thickness in these regions.

We included a large cross-sectional sample (*n* = 293) of participants ranging in age from 7 to 26 years and a slightly modified version of the computerized director task to test the reproducibility of a previously reported pattern of age-related improvements in social cognition across adolescence (Dumontheil, Apperly, et al., 2010; Symeonidou et al., 2016). Accurate performance in the experimental condition of this task is thought to depend upon use of the ability to represent what another person can see, which is a core component of theory of mind (Dumontheil, Apperly, et al., 2010; Flavell, Everett, Croft, & Flavell, 1981). The results showed that for trials specifically requiring participants to take into account the director’s perspective to identify and select the instructed target objects in cartoon pictures of a set of shelves containing multiple different objects, children made more errors than adolescents, and adolescents made more errors than adults. In comparison, for trials that required participants to follow a simple visual rule, but which were otherwise identical to the perspective taking trials, children made more errors than other group, but the accuracy of adolescents and adults did not differ.

In the original study on age-related differences in performance on the director task, Dumontheil et al. (2010) reported on data from 177 female participants 7–27 years old and similarly found that, on critical trials in the experimental condition, but not in the control condition, both children and adolescents made more errors than adults. This main finding has been replicated in two smaller studies including both male and female participants: one with 65 participants aged 9–29 years (Symeonidou et al., 2016) and one with 90 participants aged 11–18 years (Humphrey & Dumontheil, 2016). Our results also replicate this main finding and thus support the conclusion that developmental changes in use of social perspective taking are still occurring across adolescence. A caveat is that the director in our task was an older man. If social perspective taking usage is contingent on participants’ relationship with the target, it is possible that younger people are less inclined to take the perspective of this older individual. However, a previous study used a director task version with a younger director (Symeonidou et al., 2016) and found similar developmental differences as the original study (Dumontheil, Apperly, et al., 2010).

Our results for response times showed that both adolescents and adults were slower in the director condition than in the no-director condition, and counterintuitively that participants overall also were slower on control trials than on critical trials. Response time is not a key measure of interest in the director task (Humphrey & Dumontheil, 2016), and previous studies have reported conflicting findings. Inconsistent with the current results, Dumontheil et al. (2010) found slower responses in the no-director condition than in the director condition, but consistent with the current results, slower responses in control trials than in critical trials. In contrast, Humphrey and Dumontheil (2016) found no significant effects of condition or trial type, while Symeonidou et al. (2016) found a three-way interaction between condition, trial type and age.

There were some notable differences between the paradigm used in our study and that used in previous studies. Compared to previous studies, we used a modified task with simplified instructions, which did not require participants to make directional decisions. Possibly as a function of this, as the instructions were simpler, the error rates were on average much lower in our study than in previous studies (Dumontheil, Apperly, et al., 2010; Humphrey & Dumontheil, 2016; Symeonidou et al., 2016). Despite this difference, the overall pattern of age-related effects on accuracy was the same in our study as in previous studies. As the use of social perspective taking is a key component of theory of mind, these findings are also consistent with studies indicating ongoing development of mentalizing about both emotions and actions throughout adolescence (Keulers, Evers, Stiers, & Jolles, 2010; Sebastian et al., 2012; Vetter, Altgassen, Phillips, Mahy, & Kliegel, 2013; Vetter, Leipold, Kliegel, Phillips, & Altgassen, 2013). To understand the underlying factors in director task performance, Symeonidou et al. (2016) analyzed eye-tracking data acquired during correct and incorrect trials separately and found that children, adolescents, and young adults did not significantly differ in their online processing during perspective taking. This might suggest that the age-related differences in behavior are in the likelihood, rather than the nature, of perspective taking, that is, they are quantitative rather than qualitative. Other studies have investigated the possibility that inhibitory control, by allowing participants to inhibit their own perspective in favor of another individual’s perspective, may underlie developmental changes in social perspective taking. These studies have found, both in adults and in developmental samples, a positive correlation between inhibition and perspective taking (Nilsen & Graham, 2009). For instance, one study found that inhibitory control, as measured by go/no-go task performance, partly accounted for director task accuracy in adolescents (Symeonidou et al., 2016), although this finding was not replicated in a later study with a smaller age range (Humphrey & Dumontheil, 2016).

The main objective of the present study was to connect multiple levels of analysis by relating the experimental measure of social perspective taking obtained through the director task to real life social behavior and to individual differences in brain structure during adolescence. For these analyses, we used the difference in percentage errors on critical trials in the director condition and the control condition, respectively, as our measure of interest to specifically focus on social perspective taking, while controlling for general and executive function task demands.

First, supporting our hypothesis, we found that, independent of age and thus possibly indicative of developmental variability, there was a negative association between errors specifically on trials requiring use of social perspective taking and self-reported prosocial behavior. The strength of this relationship was small, but it fits with numerous studies documenting a small positive association between children’s theory of mind scores and various measures of prosocial behavior (Imuta et al., 2016). We had also hypothesized that better use of social perspective taking would be associated with fewer reported peer relationships problems, and although there was such a trend, this association was not significant. Future studies with more in-depth assessment of social behavior, including reports from multiple informants or observational data for example, should investigate this further.

Few previous studies have focused on the pro- and antisocial behavioral relevance of perspective taking ability in adolescents. One notable exception is an experimental study by Fett et al. (2014), which found that individual differences in adolescents’ social perspective taking, as measured with the director task, was associated with social behavior, specifically behavioral measures of initial trust and reciprocity in the trust game. Another exception is a study by Güroğlu et al. (2014), which found that older adolescents compared to younger adolescents showed increased differentiation in prosocial behavior depending on the relation with the interacting partner in the task (friend, antagonist, neutral classmate, or anonymous peer). Furthermore, the age-related increase in noncostly prosocial behavior toward friends was mediated by self-reported perspective taking skills (Güroğlu et al., 2014). The current study adds to this literature, by showing that adolescents’ use of social perspective taking to guide decisions and behavior is associated with more reported prosocial behavior.

Second, the current study provided novel results regarding the brain structural correlates of the use of social perspective taking. Specifically, we studied both cortical thickness and surface area, as these separate components of cortical structure have heterogeneous phylogenetic development and ontogenetic origins (Geschwind & Rakic, 2013) and distinct genetic influences and patterning (Chen et al., 2013), and critically, develop differently from childhood to adulthood (Raznahan et al., 2011; Tamnes et al., 2017; Vijayakumar et al., 2016; Wierenga et al., 2014). The results showed that, when age was not statistically controlled for, better performance specifically on trials requiring use of social perspective taking was associated with thinner cortex in widespread bilateral fronto-parietal and left hemisphere lateral temporal regions. Interestingly, among the regions showing the strongest associations were the medial prefrontal, lateral prefrontal, and anterior cingulate cortices; brain regions that have been implicated in social cognition (Apps, Rushworth, & Chang, 2016; Kilford, Garrett, & Blakemore, 2016; Schurz, Radua, Aichhorn, Richlan, & Perner, 2014; Van Overwalle, 2009) and/or executive functioning (Crone & Steinbeis, 2017; Paus, 2001; Yuan & Raz, 2014). Our results indicate temporal co-occurrence of developmental trends in use of social perspective taking and cortical thickness in these widespread regions, but these results are not sufficient evidence for directly linking the two. We therefore repeated the analyses while additionally controlling for age (together with sex), as such age-independent associations might partly be mediated by individual developmental variability.

Independent of age and sex, better performance specifically on trials requiring use of social perspective taking was associated with thinner cortex in the left hemisphere in parts of the caudal middle frontal and precentral gyri, and in a lateral parietal region covering parts of the superior and inferior parietal lobules and the postcentral gyrus. As the cerebral cortex generally, as well as in these regions specifically, decreases with age across adolescence (Tamnes et al., 2017), this might indicate that individuals with better ability to use social perspective taking have relatively more mature cortical structure in these regions. It should be noted that these results showed small effect sizes. However, age-independent relationships between behavioral and brain measures are typically moderate, likely related to the fact that there is much individual variance at both levels at any given age, and that the relationships may also fluctuate with age (Walhovd et al., 2014). Moreover, a central tenet is that the shape of brain developmental trajectories may be more strongly related to behavioral and functional characteristics than absolute brain measures at any given point during development (Giedd & Rapoport, 2010), and longitudinal studies should therefore be performed. Mediation analyses in the present cross-sectional sample did reveal indirect effects of age on social perspective taking usage via cortical thickness in these frontal and parietal regions. This supports the purported relevance of cortical development for development of social perspective taking.

Although, as far as we know, the present study is the first to investigate the brain structural correlates of social perspective taking, results from fMRI studies of adults performing a version of the director task have shown that using social perspective taking is associated with linked activation of lateral temporal cortices, and medial and lateral prefrontal regions, that is, regions typically involved in both social cognition and executive functions (Dumontheil, Küster, Apperly, & Blakemore, 2010; Hillebrandt, Dumontheil, Blakemore, & Roiser, 2013). Results from another fMRI study suggest that adolescents show greater activation in dorsal medial prefrontal cortex whenever social information is present, whereas adults only show such increased activation when the social information is relevant to task performance, and this might indicate a lesser functional specificity of this brain region in adolescence (Dumontheil, Hillebrandt, Apperly, & Blakemore, 2012). Complementing these findings, the current study link social perspective taking ability and brain structure. In contrast to the associations found between Director task performance and cortical thickness, no associations were found with cortical surface area. This might possibly relate to that this dimension of cortical structure changes less than cortical thickness across adolescence (Jernigan et al., 2016; Tamnes et al., 2017).

The current findings should be interpreted in light of the following issues. First, the results were obtained using cross-sectional data. The development of social perspective taking usage and its links with social behavior and brain structure should be further investigated with longitudinal data. Second, an important question is whether errors in the experimental condition of the director task actually reflect failure to use social perspective taking, which involves some understanding of another person’s preferences, goals, intentions and so forth, or selective attention (Rubio-Fernandez, 2017) or visuospatial manipulation failure (Fett et al., 2014). Studies of adults indicate that errors on this type of task do not arise simply as a result of failure to effectively switch perspectives (Apperly et al., 2010), but further studies on developmental samples comparing visuospatial processing abilities and performance on the director task are called for. Third, and related to the previous issue, our results showed relationships between use of social perspective taking and cortical thickness in regions including the superior parietal lobule and the caudal middle frontal gyrus, regions known to show increased activity associated with visual perspective taking (Schurz, Aichhorn, Martin, & Perner, 2013) and mental rotation (Tomasino & Gremese, 2016). This also begs the question as to what degree the employed task really requires social perspective taking. However, a growing body of work links the visuospatial and the social aspects of perspective taking (Hamilton, Kessler, & Creem-Regehr, 2014) and it can thus be argued against a simple distinction between the two. Nonetheless, future neuroimaging studies are needed to investigate to what degree the two are dissociable in terms of brain structure and function. Finally, the assessment of prosocial behavior and peer relationship problems was limited to brief self-report questionnaire scales.

There has recently been a call for more large-scale studies on individual differences in neurocognitive development (Foulkes & Blakemore, 2018). The results of the current study, which used an experimental approach and a large cross-sectional sample of participants aged 7–26 years, replicate the findings of earlier studies indicating continued development of use of social perspective taking across adolescence. Furthermore, within subsamples, the study yielded novel results linking individual differences in use of social perspective taking with a higher level of real-life prosocial behavior and with thinner and possibly more mature cerebral cortex in fronto-parietal regions.

## Supplementary Material

10.1037/dev0000541.supp

## Figures and Tables

**Table 1 tbl1:** Task Performance Summary

Task	Errors (%)		Response time (ms)	
	*M*	*SD*	*M*	*SD*
Director filler trials	1.6	3.3	2211.1	353.5
Director control trials	3.0	6.9	2851.2	616.8
Director critical trials	13.2	23.9	2664.4	632.4
No-director filler trials	1.4	2.7	2211.8	402.4
No-director control trials	1.5	4.9	2723.9	633.4
No-director critical trials	4.4	10.6	2594.1	648.2
Difference director critical–no-director critical	8.8	21.6		
*Note*. *n* = 293.				

## References

[c1] Apperly I. A., Carroll D. J., Samson D., Humphreys G. W., Qureshi A., & Moffitt G. (2010). Why are there limits on theory of mind use? Evidence from adults’ ability to follow instructions from an ignorant speaker. The Quarterly Journal of Experimental Psychology, 63, 1201–1217. 10.1080/17470210903281582 19838912

[c2] Apps M. A., Rushworth M. F., & Chang S. W. (2016). The anterior cingulate gyrus and social cognition: Tracking the motivation of others. Neuron, 90, 692–707. 10.1016/j.neuron.2016.04.018 27196973 PMC4885021

[c3] Austin G., Bondü R., & Elsner B. (2017). Longitudinal relations between children’s cognitive and affective theory of mind with reactive and proactive aggression. Aggressive Behavior, 43, 440–449. 10.1002/ab.21702 28217944

[c4] Begeer S., Bernstein D. M., Aßfalg A., Azdad H., Glasbergen T., Wierda M., & Koot H. M. (2016). Equal egocentric bias in school-aged children with and without autism spectrum disorders. Journal of Experimental Child Psychology, 144, 15–26. 10.1016/j.jecp.2015.10.018 26687336

[c5] Blakemore S. J., & Mills K. L. (2014). Is adolescence a sensitive period for sociocultural processing? Annual Review of Psychology, 65, 187–207. 10.1146/annurev-psych-010213-115202 24016274

[c6] Blakemore S. J., & Robbins T. W. (2012). Decision-making in the adolescent brain. Nature Neuroscience, 15, 1184–1191. 10.1038/nn.3177 22929913

[c7] Braams B. R., & Crone E. A. (2017). Longitudinal changes in social brain development: Processing outcomes for friend and self. Child Development, 88, 1952–1965. 10.1111/cdev.12665 27861755

[c8] Caputi M., Lecce S., Pagnin A., & Banerjee R. (2012). Longitudinal effects of theory of mind on later peer relations: The role of prosocial behavior. Developmental Psychology, 48, 257–270. 10.1037/a0025402 21895361

[c9] Chen C. H., Fiecas M., Gutiérrez E. D., Panizzon M. S., Eyler L. T., Vuoksimaa E., . . . Kremen W. S. (2013). Genetic topography of brain morphology. Proceedings of the National Academy of Sciences of the United States of America, 110, 17089–17094. 10.1073/pnas.1308091110 24082094 PMC3801007

[c10] Crone E. A., & Steinbeis N. (2017). Neural perspectives on cognitive control development during childhood and adolescence. Trends in Cognitive Sciences, 21, 205–215. 10.1016/j.tics.2017.01.003 28159355

[c11] Cullen H., Kanai R., Bahrami B., & Rees G. (2014). Individual differences in anthropomorphic attributions and human brain structure. Social Cognitive and Affective Neuroscience, 9, 1276–1280. 10.1093/scan/nst109 23887807 PMC4158361

[c12] Dale A. M., Fischl B., & Sereno M. I. (1999). Cortical surface-based analysis. I. Segmentation and surface reconstruction. NeuroImage, 9, 179–194. 10.1006/nimg.1998.0395 9931268

[c13] Decety J., Bartal I. B., Uzefovsky F., & Knafo-Noam A. (2016). Empathy as a driver of prosocial behaviour: Highly conserved neurobehavioural mechanisms across species. Philosophical Transactions of the Royal Society of London Series B: Biological Sciences, 371, 20150077 10.1098/rstb.2015.0077 26644596 PMC4685523

[c14] Dumontheil I., Apperly I. A., & Blakemore S. J. (2010). Online usage of theory of mind continues to develop in late adolescence. Developmental Science, 13, 331–338. 10.1111/j.1467-7687.2009.00888.x 20136929

[c15] Dumontheil I., Hillebrandt H., Apperly I. A., & Blakemore S. J. (2012). Developmental differences in the control of action selection by social information. Journal of Cognitive Neuroscience, 24, 2080–2095. 10.1162/jocn_a_00268 22784276

[c16] Dumontheil I., Küster O., Apperly I. A., & Blakemore S. J. (2010). Taking perspective into account in a communicative task. NeuroImage, 52, 1574–1583. 10.1016/j.neuroimage.2010.05.056 20510369

[c17] Fett A. K., Shergill S. S., Gromann P. M., Dumontheil I., Blakemore S. J., Yakub F., & Krabbendam L. (2014). Trust and social reciprocity in adolescence—a matter of perspective-taking. Journal of Adolescence, 37, 175–184. 10.1016/j.adolescence.2013.11.011 24439623

[c18] Fischl B. (2012). FreeSurfer. NeuroImage, 62, 774–781. 10.1016/j.neuroimage.2012.01.021 22248573 PMC3685476

[c19] Fischl B., & Dale A. M. (2000). Measuring the thickness of the human cerebral cortex from magnetic resonance images. Proceedings of the National Academy of Sciences of the United States of America, 97, 11050–11055. 10.1073/pnas.200033797 10984517 PMC27146

[c20] Fischl B., Salat D. H., Busa E., Albert M., Dieterich M., Haselgrove C., . . . Dale A. M. (2002). Whole brain segmentation: Automated labeling of neuroanatomical structures in the human brain. Neuron, 33, 341–355. 10.1016/S0896-6273(02)00569-X 11832223

[c21] Fischl B., Sereno M. I., & Dale A. M. (1999). Cortical surface-based analysis. II: Inflation, flattening, and a surface-based coordinate system. NeuroImage, 9, 195–207. 10.1006/nimg.1998.0396 9931269

[c22] Flannery J. E., Giuliani N. R., Flournoy J. C., & Pfeifer J. H. (2017). Neurodevelopmental changes across adolescence in viewing and labeling dynamic peer emotions. Developmental Cognitive Neuroscience, 25, 113–127. 10.1016/j.dcn.2017.02.003 28262423 PMC5764159

[c23] Flavell J. H., Everett B. A., Croft K., & Flavell E. R. (1981). Young children’s knowledge about visual perception: Further evidence for the Level 1–Level 2 distinction. Developmental Psychology, 17, 99–103. 10.1037/0012-1649.17.1.99

[c24] Flournoy J. C., Pfeifer J. H., Moore W. E., Tackman A. M., Masten C. L., Mazziotta J. C., . . . Dapretto M. (2016). Neural reactivity to emotional faces may mediate the relationship between childhood empathy and adolescent prosocial behavior. Child Development, 87, 1691–1702. 10.1111/cdev.12630 28262939 PMC5448462

[c25] Foulkes L., & Blakemore S. J. (2018). Studying individual differences in human adolescent brain development. Nature Neuroscience, 21, 315–323. 10.1038/s41593-018-0078-4 29403031

[c26] Geschwind D. H., & Rakic P. (2013). Cortical evolution: Judge the brain by its cover. Neuron, 80, 633–647. 10.1016/j.neuron.2013.10.045 24183016 PMC3922239

[c27] Giedd J. N., & Rapoport J. L. (2010). Structural MRI of pediatric brain development: What have we learned and where are we going? Neuron, 67, 728–734. 10.1016/j.neuron.2010.08.040 20826305 PMC3285464

[c28] Giedd J. N., Raznahan A., Alexander-Bloch A., Schmitt E., Gogtay N., & Rapoport J. L. (2015). Child psychiatry branch of the National Institute of Mental Health longitudinal structural magnetic resonance imaging study of human brain development. Neuropsychopharmacology, 40, 43–49. 10.1038/npp.2014.236 25195638 PMC4262916

[c29] Goodman A., & Goodman R. (2009). Strengths and difficulties questionnaire as a dimensional measure of child mental health. Journal of the American Academy of Child & Adolescent Psychiatry, 48, 400–403. 10.1097/CHI.0b013e3181985068 19242383

[c30] Goodman R., Meltzer H., & Bailey V. (1998). The Strengths and Difficulties Questionnaire: A pilot study on the validity of the self-report version. European Child & Adolescent Psychiatry, 7, 125–130. 10.1007/s007870050057 9826298

[c31] Güroğlu B., van den Bos W., & Crone E. A. (2014). Sharing and giving across adolescence: An experimental study examining the development of prosocial behavior. Frontiers in Psychology, 5, 291. 24782796 10.3389/fpsyg.2014.00291 PMC3990099

[c32] Hagler D. J. Jr., Saygin A. P., & Sereno M. I. (2006). Smoothing and cluster thresholding for cortical surface-based group analysis of fMRI data. NeuroImage, 33, 1093–1103. 10.1016/j.neuroimage.2006.07.036 17011792 PMC1785301

[c33] Hamilton A. F., Kessler K., & Creem-Regehr S. H. (2014). Perspective taking: Building a neurocognitive framework for integrating the “social” and the “spatial.” Frontiers in Human Neuroscience, 8, 403 10.3389/fnhum.2014.00403 24966824 PMC4052522

[c34] Hayasaka S., & Nichols T. E. (2003). Validating cluster size inference: Random field and permutation methods. NeuroImage, 20, 2343–2356. 10.1016/j.neuroimage.2003.08.003 14683734

[c35] Hayes A. F. (2013). Introduction to mediation, moderation, and conditional process analysis: A regression-based approach. New York, NY: Guilford Press.

[c36] Hillebrandt H., Dumontheil I., Blakemore S. J., & Roiser J. P. (2013). Dynamic causal modelling of effective connectivity during perspective taking in a communicative task. NeuroImage, 76, 116–124. 10.1016/j.neuroimage.2013.02.072 23507383

[c37] Humphrey G., & Dumontheil I. (2016). Development of risk-taking, perspective-taking, and inhibitory control during adolescence. Developmental Neuropsychology, 41, 59–76. 10.1080/87565641.2016.1161764 27070826

[c38] Imuta K., Henry J. D., Slaughter V., Selcuk B., & Ruffman T. (2016). Theory of mind and prosocial behavior in childhood: A meta-analytic review. Developmental Psychology, 52, 1192–1205. 10.1037/dev0000140 27337508

[c39] Jernigan T. L., Baaré W. F., Stiles J., & Madsen K. S. (2011). Postnatal brain development. Progress in Brain Research, 189, 77–92. 10.1016/B978-0-444-53884-0.00019-1 21489384 PMC3690327

[c40] Jernigan T. L., Brown T. T., Bartsch H., & Dale A. M. (2016). Toward an integrative science of the developing human mind and brain: Focus on the developing cortex. Developmental Cognitive Neuroscience, 18, 2–11. 10.1016/j.dcn.2015.07.008 26347228 PMC4762760

[c41] Kanai R., Bahrami B., Roylance R., & Rees G. (2012). Online social network size is reflected in human brain structure. Proceedings of the Royal Society B: Biological Sciences, 279, 1327–1334. 10.1098/rspb.2011.1959 22012980 PMC3282379

[c42] Kanai R., & Rees G. (2011). The structural basis of inter-individual differences in human behaviour and cognition. Nature Reviews Neuroscience, 12, 231–242. 10.1038/nrn3000 21407245

[c43] Keulers E. H., Evers E. A., Stiers P., & Jolles J. (2010). Age, sex, and pubertal phase influence mentalizing about emotions and actions in adolescents. Developmental Neuropsychology, 35, 555–569. 10.1080/87565641.2010.494920 20721775

[c44] Keysar B., Barr D. J., Balin J. A., & Brauner J. S. (2000). Taking perspective in conversation: The role of mutual knowledge in comprehension. Psychological Science, 11, 32–38. 10.1111/1467-9280.00211 11228840

[c45] Keysar B., Lin S., & Barr D. J. (2003). Limits on theory of mind use in adults. Cognition, 89, 25–41. 10.1016/S0010-0277(03)00064-7 12893123

[c46] Kilford E. J., Garrett E., & Blakemore S. J. (2016). The development of social cognition in adolescence: An integrated perspective. Neuroscience and Biobehavioral Reviews, 70, 106–120. 10.1016/j.neubiorev.2016.08.016 27545755

[c47] Krogsrud S. K., Tamnes C. K., Fjell A. M., Amlien I., Grydeland H., Sulutvedt U., . . . Walhovd K. B. (2014). Development of hippocampal subfield volumes from 4 to 22 years. Human Brain Mapping, 35, 5646–5657. 10.1002/hbm.22576 24976170 PMC6869672

[c48] Luna B., Padmanabhan A., & O’Hearn K. (2010). What has fMRI told us about the development of cognitive control through adolescence? Brain and Cognition, 72, 101–113. 10.1016/j.bandc.2009.08.005 19765880 PMC2815087

[c49] Lyall A. E., Shi F., Geng X., Woolson S., Li G., Wang L., . . . Gilmore J. H. (2015). Dynamic development of regional cortical thickness and surface area in early childhood. Cerebral Cortex, 25, 2204–2212. 10.1093/cercor/bhu027 24591525 PMC4506327

[c50] Mills K. L., Dumontheil I., Speekenbrink M., & Blakemore S. J. (2015). Multitasking during social interactions in adolescence and early adulthood. Royal Society Open Science, 2, 150117 10.1098/rsos.150117 26715991 PMC4680606

[c51] Mills K. L., Lalonde F., Clasen L. S., Giedd J. N., & Blakemore S. J. (2014). Developmental changes in the structure of the social brain in late childhood and adolescence. Social Cognitive and Affective Neuroscience, 9, 123–131. 10.1093/scan/nss113 23051898 PMC3871734

[c52] Mills K. L., & Tamnes C. K. (2014). Methods and considerations for longitudinal structural brain imaging analysis across development. Developmental Cognitive Neuroscience, 9, 172–190. 10.1016/j.dcn.2014.04.004 24879112 PMC6989768

[c53] Moll H., & Tomasello M. (2006). Level 1 perspective-taking at 24 months of age. British Journal of Developmental Psychology, 24, 603–613. 10.1348/026151005X55370

[c54] Nilsen E. S., & Graham S. A. (2009). The relations between children’s communicative perspective-taking and executive functioning. Cognitive Psychology, 58, 220–249. 10.1016/j.cogpsych.2008.07.002 18809176

[c55] Overgaauw S., van Duijvenvoorde A. C., Gunther Moor B., & Crone E. A. (2015). A longitudinal analysis of neural regions involved in reading the mind in the eyes. Social Cognitive and Affective Neuroscience, 10, 619–627. 10.1093/scan/nsu095 25062837 PMC4420738

[c56] Paus T. (2001). Primate anterior cingulate cortex: Where motor control, drive and cognition interface. Nature Reviews Neuroscience, 2, 417–424. 10.1038/35077500 11389475

[c57] Perner J., & Wimmer H. (1985). “John thinks that Mary thinks that . . .” Attributions of second-order beliefs by 5- to 10-year-old children. Journal of Experimental Child Psychology, 39, 437–471. 10.1016/0022-0965(85)90051-7

[c58] Raznahan A., Shaw P., Lalonde F., Stockman M., Wallace G. L., Greenstein D., . . . Giedd J. N. (2011). How does your cortex grow? The Journal of Neuroscience, 31, 7174–7177. 10.1523/JNEUROSCI.0054-11.2011 21562281 PMC3157294

[c59] Rubio-Fernández P. (2017). The director task: A test of theory-of-mind use or selective attention? Psychonomic Bulletin & Review, 24, 1121–1128. 10.3758/s13423-016-1190-7 27822775

[c60] Salthouse T. A. (2011). Neuroanatomical substrates of age-related cognitive decline. Psychological Bulletin, 137, 753–784. 10.1037/a0023262 21463028 PMC3132227

[c61] Schurz M., Aichhorn M., Martin A., & Perner J. (2013). Common brain areas engaged in false belief reasoning and visual perspective taking: A meta-analysis of functional brain imaging studies. Frontiers in Human Neuroscience, 7, 712 10.3389/fnhum.2013.00712 24198773 PMC3814428

[c62] Schurz M., Radua J., Aichhorn M., Richlan F., & Perner J. (2014). Fractionating theory of mind: A meta-analysis of functional brain imaging studies. Neuroscience and Biobehavioral Reviews, 42, 9–34. 10.1016/j.neubiorev.2014.01.009 24486722

[c63] Sebastian C. L., Fontaine N. M. G., Bird G., Blakemore S.-J., De Brito S. A., McCrory E. J., & Viding E. (2012). Neural processing associated with cognitive and affective theory of mind in adolescents and adults. Social Cognitive and Affective Neuroscience, 7, 53–63. 10.1093/scan/nsr023 21467048 PMC3252629

[c64] Sierksma J., Thijs J., Verkuyten M., & Komter A. (2014). Children’s reasoning about the refusal to help: The role of need, costs, and social perspective taking. Child Development, 85, 1134–1149. 10.1111/cdev.12195 24936613

[c65] Sodian B., Thoermer C., & Metz U. (2007). Now I see it but you don’t: 14-month-olds can represent another person’s visual perspective. Developmental Science, 10, 199–204. 10.1111/j.1467-7687.2007.00580.x 17286844

[c66] Storsve A. B., Fjell A. M., Tamnes C. K., Westlye L. T., Øverbye K., Aasland H. W., & Walhovd K. B. (2014). Differential longitudinal changes in cortical thickness, surface area and volume across the adult life span: Regions of accelerating and decelerating change. The Journal of Neuroscience, 34, 8488–8498. 10.1523/JNEUROSCI.0391-14.2014 24948804 PMC6608217

[c67] Symeonidou I., Dumontheil I., Chow W. Y., & Breheny R. (2016). Development of online use of theory of mind during adolescence: An eye-tracking study. Journal of Experimental Child Psychology, 149, 81–97. 10.1016/j.jecp.2015.11.007 26723471

[c68] Tamnes C. K., Herting M. M., Goddings A. L., Meuwese R., Blakemore S. J., Dahl R. E., . . . Mills K. L. (2017). Development of the cerebral cortex across adolescence: A multisample study of inter-related longitudinal changes in cortical volume, surface area, and thickness. The Journal of Neuroscience, 37, 3402–3412. 10.1523/JNEUROSCI.3302-16.2017 28242797 PMC5373125

[c69] Tamnes C. K., Østby Y., Fjell A. M., Westlye L. T., Due-Tønnessen P., & Walhovd K. B. (2010). Brain maturation in adolescence and young adulthood: Regional age-related changes in cortical thickness and white matter volume and microstructure. Cerebral Cortex, 20, 534–548. 10.1093/cercor/bhp118 19520764

[c70] Tamnes C. K., Walhovd K. B., Dale A. M., Østby Y., Grydeland H., Richardson G., . . . the Alzheimer’s Disease Neuroimaging Initiative (2013). Brain development and aging: Overlapping and unique patterns of change. NeuroImage, 68, 63–74. 10.1016/j.neuroimage.2012.11.039 23246860 PMC5378867

[c71] Tomasino B., & Gremese M. (2016). Effects of stimulus type and strategy on mental rotation network: An activation likelihood estimation meta-analysis. Frontiers in Human Neuroscience, 9, 693 10.3389/fnhum.2015.00693 26779003 PMC4704562

[c72] Van Overwalle F. (2009). Social cognition and the brain: A meta-analysis. Human Brain Mapping, 30, 829–858. 10.1002/hbm.20547 18381770 PMC6870808

[c73] Vetter N. C., Altgassen M., Phillips L., Mahy C. E., & Kliegel M. (2013). Development of affective theory of mind across adolescence: Disentangling the role of executive functions. Developmental Neuropsychology, 38, 114–125. 10.1080/87565641.2012.733786 23410214

[c74] Vetter N. C., Leipold K., Kliegel M., Phillips L. H., & Altgassen M. (2013). Ongoing development of social cognition in adolescence. Child Neuropsychology, 19, 615–629. 10.1080/09297049.2012.718324 22934659

[c75] Vijayakumar N., Allen N. B., Youssef G., Dennison M., Yücel M., Simmons J. G., & Whittle S. (2016). Brain development during adolescence: A mixed-longitudinal investigation of cortical thickness, surface area, and volume. Human Brain Mapping, 37, 2027–2038. 10.1002/hbm.23154 26946457 PMC6867680

[c76] Von Der Heide R., Vyas G., & Olson I. R. (2014). The social network-network: Size is predicted by brain structure and function in the amygdala and paralimbic regions. Social Cognitive and Affective Neuroscience, 9, 1962–1972. 10.1093/scan/nsu009 24493846 PMC4249478

[c77] Walhovd K. B., Tamnes C. K., & Fjell A. M. (2014). Brain structural maturation and the foundations of cognitive behavioral development. Current Opinion in Neurology, 27, 176–184. 10.1097/WCO.0000000000000074 24565941

[c78] Wechsler D. (1999). Wechsler Abbreviated Scale of Intelligence (WASI). San Antonio, TX: The Psychological Corporation.

[c79] Wellman H. M., Cross D., & Watson J. (2001). Meta-analysis of theory-of-mind development: The truth about false belief. Child Development, 72, 655–684. 10.1111/1467-8624.00304 11405571

[c80] Wierenga L. M., Langen M., Oranje B., & Durston S. (2014). Unique developmental trajectories of cortical thickness and surface area. NeuroImage, 87, 120–126. 10.1016/j.neuroimage.2013.11.010 24246495

[c81] Yuan P., & Raz N. (2014). Prefrontal cortex and executive functions in healthy adults: A meta-analysis of structural neuroimaging studies. Neuroscience and Biobehavioral Reviews, 42, 180–192. 10.1016/j.neubiorev.2014.02.005 24568942 PMC4011981

