# Supplementary material for: Social Perspective Taking Is Associated With Self-Reported Prosocial Behavior and Regional Cortical Thickness Across Adolescence
Source: Dev Psychol. 2018 Jul 30;54(9):1745–57. doi: 10.1037/dev0000541 (PMC6110335; doi:10.1037/dev0000541)
Supplement: Supplementary file 1 [file Supplemental-Materials_dev0000541.docx]

**Social perspective taking is associated with self-reported prosocial behavior and regional cortical thickness across adolescence**

**Supplemental Material**

**Materials and Methods**

***Statistical analysis***

To test whether the age-related differences found in the analyses using age groups held when treating age as a continuous variable, additional analyses were performed. For both accuracy and RT, we ran a mixed ANOVA with Condition (Director, No-Director) and Trial type (Critical, Control) as within-subject factors and age (standardized) as a covariate. Follow-up analyses were performed to further investigate significant interaction and main effects.

**Results**

***Director task: Accuracy***

All main effects were significant in a mixed ANOVA on accuracy with Condition (Director, No-Director) and Trial type (Critical, Control) as within-subject factors and age as covariate. Participants made more errors in the Director condition than in the No-Director condition (F(1,291) = 60.40, p < .001, η*_p_*^2^ = .172), more errors on Critical trials than on Control trials (F(1,291) = 69.49, p < .001, η*_p_*^2^ = .193), and accuracy showed an age-related improvement (F(1,291) = 80.02, p < .001, η*_p_*^2^ = .216). There was a significant interaction between Condition and Trial type (F(1,291) = 30.47, p < .001, η*_p_*^2^ = .095), between Condition and age (F(1,291) = 14.33, p < .001, η*_p_*^2^ = .047), and between Trial type and age (F(1,291) = 38.54, p < .001, η*_p_*^2^ = .117). The three-way interaction was also significant (F(1,291) = 10.71, p = .001, η*_p_*^2^ = .035), and was explored further by looking at Critical and Control trials separately.

A mixed ANOVA performed on the Critical trials showed main effects of Condition (F(1,291) = 50.42, p < .001, η*_p_*^2^ = .148), with more errors in the Director condition, and age, with decreasing percentage errors with age (F(1,291) = 64.04, p < .001, η*_p_*^2^ = .180), as well as a significant interaction between Condition and age (F(1,291) = 14.22, p < .001, η*_p_*^2^ = .047). The same analysis on the Control trials only also showed significant main effects of Condition (F(1,291) = 9.98, p = .002, η*_p_*^2^ = .033) and age (F(1,291) = 34.44, p < .001, η*_p_*^2^ = .106), but no significant interaction effect (F(1,291) = 0.51, p = .478, η*_p_*^2^ = .002).

Analyses on the Critical trials in the two conditions separately showed a significant decrease in accuracy with age in both the Director condition (F(1,291) = 44.46, p < .001, η*_p_*^2^ = .133) and the No-Director condition (F(1,291) = 49.47, p < .001, η*_p_*^2^ = .145). A follow-up analysis on the difference in percentage errors on Director Critical trials and on No-Director Critical trials showed a significant effect of age (F(1,291) = 14.22, p < .001, η*_p_*^2^ = .047), i.e. a steeper slope corresponding to greater improvement in performance with age in Director Critical than No-Director Critical trials.

***Director task: Response times***

In a mixed ANOVA on RT with Condition (Director, No-Director) and Trial type (Critical, Control) as within-subject factors and age as covariate, the main effects of Condition (F(1,291) = 18.81, p < .001, η*_p_*^2^ = .061), Trial type (F(1,291) = 79.57, p < .001, η*_p_*^2^ = .215), and age (F(1,291) = 172.07, p < .001, η*_p_*^2^ = .372) were all significant. There was a significant interaction between Condition and age (F(1,291) = 13.80, p < .001, η*_p_*^2^ = .045), but no significant interactions between Trial type and age (F(1,291) = 1.68, p = .196, η*_p_*^2^ = .006), or Condition and Trial type (F(1,291) = 3.39, p = .067, η*_p_*^2^ = .012). The three-way interaction was not significant (F(1,291) < 0.01, p = .996, η*_p_*^2^ < .001). Due to the interaction between Condition and age, the main effect of age on RTs was explored further in each Condition separately. The results showed a significant effect of age on RT in both the Director condition (F(1,291) = 105.43, p < .001, η*_p_*^2^ = .266) and the No-Director condition (F(1,291) = 193.60, p < .001, η*_p_*^2^ = .399), with a steeper decrease in RT with age in the No-Director condition than in the Director condition.
